# Comparison of the Efficacy and Safety of Duloxetine and Gabapentin in Diabetic Peripheral Neuropathic Pain: A Meta-Analysis

**DOI:** 10.1155/2022/4084420

**Published:** 2022-03-01

**Authors:** Lanying Jiang, Yadan Xiong, Jinguo Cui

**Affiliations:** ^1^Department of Geriatrics, Zhejiang Hospital of Integrated Traditional Chinese and Western Medicine, Hangzhou, Zhejiang, China; ^2^Department of Endocrinology and Metabolism, the 908th Hospital of Chinese People's Liberation Army Joint Logistic Support Force, Nanchang, Jiangxi, China; ^3^Department of Pharmacy, Tianjin Baodi Hospital, Baodi Clinical College of Tianjin Medical University, Tianjin, China

## Abstract

**Background:**

Diabetic peripheral neuropathic pain (DPNP) is a common chronic pain condition affecting diabetic patients and has growing importance because of the increasing prevalence of patients with type 2 diabetes mellitus. Pain is the most troublesome symptom of DPNP, increasingly recognized as an important and independent feature of DPNP. This meta-analysis aims to compare the efficacy and safety of duloxetine and gabapentin in the treatment of diabetic peripheral neuropathic pain (DPNP) and therefore to provide evidence-based medicine for clinical treatment.

**Methods:**

Relevant randomized controlled trials on duloxetine versus gabapentin for DPNP were searched from PubMed, Embase, Cochrane Library, Web of Science, CNKI, WanFang, VIP, and Chinese Biomedical Literature Database from database inception to October 2021. The data were analyzed by RevMan 5.3 software.

**Results:**

Seven studies were included. The results showed that, at the end of the study, duloxetine was significantly superior to gabapentin in terms of the incidence of adverse reactions (RR = 0.59, 95% CI: 0.45–0.79, *P* < 0.01), sleep interference score (SMD = −0.35, 95% CI: −0.63 to −0.08, *P* < 0.05), but no significant differences in VAS score (SMD = −0.14, 95% CI: −0.31–0.03, *P* > 0.05), overall response rate (RR = 1.05, 95% CI: 0.92–1.20, *P* > 0.05), and clinical global impression of change (SMD = 0.07, 95% CI: −0.20–0.35, *P* > 0.05).

**Conclusion:**

Compared with gabapentin, duloxetine has no obvious advantage in the treatment of diabetic peripheral neuralgia, but it has less side effects and significantly higher safety.

## 1. Introduction

Diabetic peripheral neuropathic pain (DPNP), with an incidence up to 26% in all diabetic patients, is one of the most common, complex, and serious complications of diabetes [1]. This disease is mainly characterized by symmetrical numbness, paresthesia, and pain in the distal extremities, with symptoms ranging from mild sensory disturbances to severe persistent pain [2]. DPNP significantly affects the patients' quality of life, and if left untreated, it may also progress to diabetic foot ulcers and even amputations. The exact pathophysiological mechanism of DPNP is unknown, so the current treatments are mainly to control pain but no approved treatment to restore neurological function [3]. In addition to glycemic control, the therapeutic base for all diabetic complications, antidepressant or anticonvulsant drugs are recommended for DPNP.

Duloxetine, originally approved for the treatment of major depression, is a selective serotonin (5-HT) and norepinephrine (NE) reuptake inhibitor (SNRI) [3]. This drug is the first SNRI for DPNP approved by the U.S. Food and Drug Administration (FDA) in September 2004 [4]. 5-HT and NE are two main neurotransmitters involving in the descending mechanism of pain; 5-HT can not only inhibit pain perception but also facilitate pain perception; NE released by peripheral sympathetic postganglionic fibers is involved in the generation of pain. Inhibition of 5-HT and NE reuptake can enhance the function of these two descending inhibitory pathways and therefore reduce ascending of nociceptive signals in the spinal cord and consequently analgesia [5].

Gabapentin, an anticonvulsant drug, was introduced as a first-line drug for a variety of neuralgia by the European Federation of Neurological Societies (EFNS) in November 2006 [6]. As a synthetic amino acid, the mechanism of its effect against allodynia includes increasing the input of inhibitors of the gamma-aminobutyric acid (GABA) mediated pathway, antagonizing N-methyl-D-aspartate (NMDA) receptors, antagonizing calcium channels in the central nervous system, and inhibiting the conduction of peripheral nerves, which is a better clinical drug for neuropathic pain [6]. Additionally, evidence shows that the mechanism of its efficacy in the treatment of diabetic neuralgia may be related to the downregulation of *α*2*δ*-1 subunit in the spinal cord [7].

Although the two drugs have been approved for many years, there are few studies on their efficacy and safety in the treatment of DPNP. Sample size in each study in this field is small, and there is no systematic review. Therefore, for expanding the sample size, this study used meta-analysis to summarize the existing studies on the advantages and disadvantages of duloxetine (with gabapentin as control) for DPNP, thus providing evidence-based medical evidence for its clinical treatment.

## 2. Methods

### 2.1. Literature Retrieval

The relevant articles were searched from Cochrane Library, PubMed, Embase, Web of Science, Chinese Biomedical Literature Database, CNKI, WanFang, and VIP. By using a combination of subject words and free words, the search terms were (“Diabetic Neuropathies” OR “Painful Diabetic Neuropathy” OR “Painful Diabetic Peripheral Polyneuropathy”) AND (“Duloxetine” OR “Cymbalta”) AND (“Gabapentin” OR “Neurontin”). Two researchers independently completed literature retrieval and then cross-checked. The search period was from database inception to October 2021, with no language restrictions.

### 2.2. Inclusion and Exclusion Criteria

#### 2.2.1. Inclusion Criteria

  Population: (1) age ≥18 years; (2) history of DPNP ≥6 months; (3) history of type 2 diabetes mellitus ≥1 year; (4) baseline visual analogue scale (VAS) score ≥40.
  Interventions: the experimental group was treated with duloxetine for DPNP.
  Comparison: the control group was treated with gabapentin for DPNP.
  Outcome measures: the primary outcome measures included VAS score and incidence of adverse reactions. Secondary outcome measures were response rate, sleep interference score, and clinical global impression of change. A study with any of the above outcome measures was included in this meta-analysis.
  Study design: randomized controlled trials (RCTs) on duloxetine versus gabapentin in DPNP.

#### 2.2.2. Exclusion Criteria

(1) Case reports, conference abstracts, and literature that could not obtain the full text; (2) course of treatment <1 month; (3) patients who were treated with other drugs or stopped taking other drugs for less than one week; (4) patients with cognitive impairment, alcoholism, or drug abuse.

### 2.3. Literature Screening and Data Extraction

Based on the abovementioned inclusion criteria and exclusion criteria, two researchers independently screened the literature and extracted the data and then cross-checked. They discussed with each other to reach an agreement on the controversial issues. The extracted data included the title, publication year, author, study design, number of study subjects, and outcome measures.

### 2.4. Quality Evaluation of Included Studies

Two researchers independently evaluated the overall quality of the included studies according to the modified Jadad scale [8], with the following items for evaluation: randomization method, allocation concealment, double-binding, incomplete outcome reporting, selective reporting bias, and other biases. The risk of bias of the literature was evaluated using RevMan 5.3 software as a tool.

### 2.5. Statistical Analysis

RevMan 5.3 software was utilized for meta-analysis, and forest plots, for calculating the pooled effect size. Heterogeneity among studies was quantitatively analyzed by *P* value and *I*^2^. In case of low heterogeneity (*P* > 0.05 and *I*^2^ < 50%), the fixed effects model was selected for meta-analysis; otherwise (*P* ≤ 0.05 or *I*^2^ ≥ 50%), the random effects model was used. When the heterogeneity among studies was high, sensitivity analysis was performed to check whether the results were robust; studies that seriously affected the heterogeneity were excluded before the analysis. Finally, funnel plots were drawn to assess the publication bias of the studies. The relative risk (RR) and its 95% confidence interval (CI) were used as the analysis statistics for dichotomous data, and the standardized mean difference (SMD) and its 95% CI for continuous data.

## 3. Results

### 3.1. Results of Literature Retrieval

**Figure 1:**
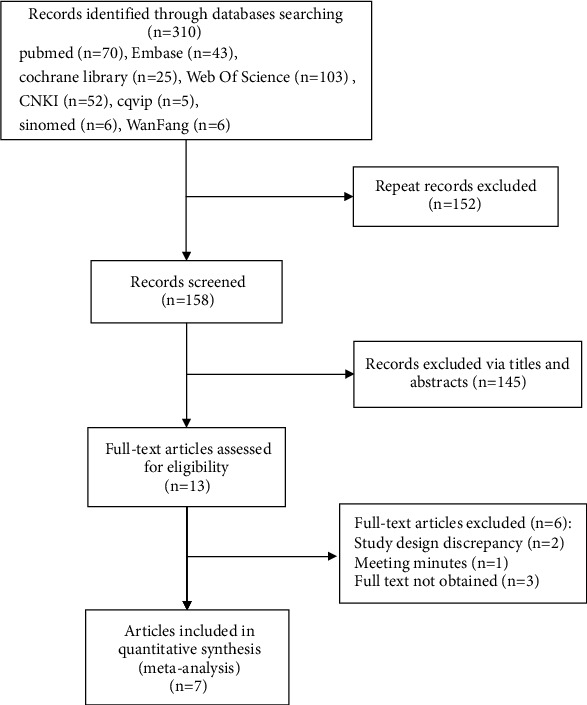
Flow graph of literature screening process.

In this meta-analysis, 310 articles were initially retrieved, and after layer-by-layer screening, seven articles [2, 7, 9–13] were finally included. All 7 articles were RCT trials, including 624 patients. The literature screening process is shown in Figure 1.

### 3.2. General Characteristics of Included Studies

In the 7 studies (624 patients), the experimental group treated with duloxetine while gabapentin in the control group; the primary outcome measures were VAS pain score and incidence of adverse reactions, and the secondary outcome measures were response rate, sleep interference score, and clinical global impression of change. The characteristics of the included studies are shown in Table 1.

### 3.3. Methodological Quality Evaluation

**Figure 2:**
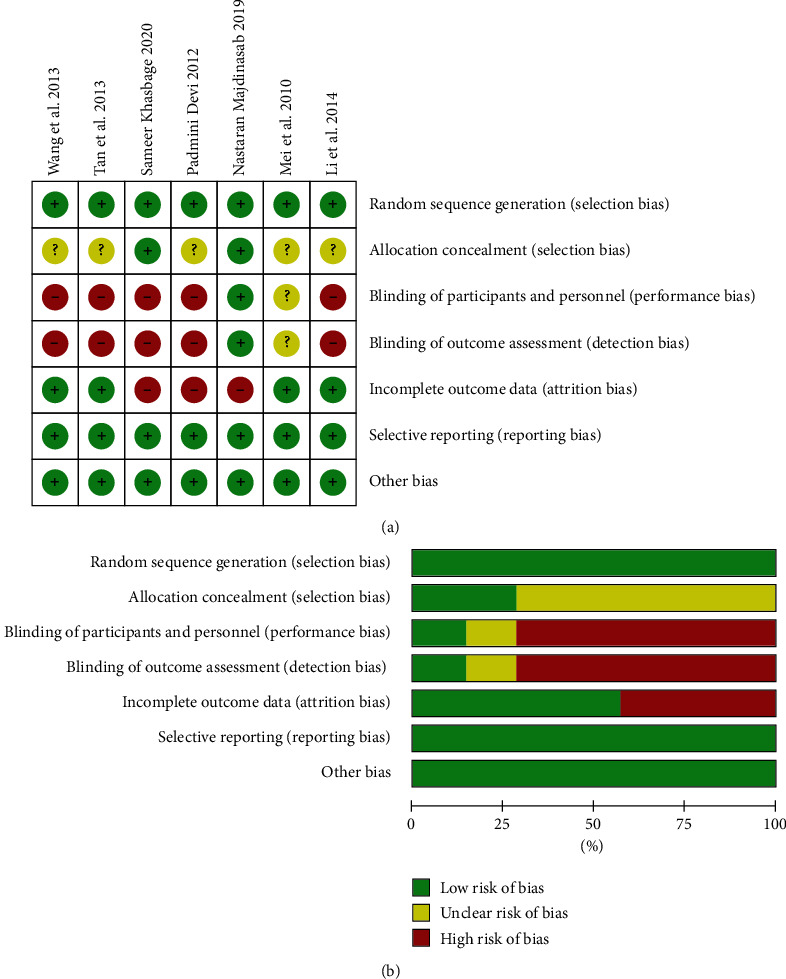
Quality evaluation of included studies. (a) A plot of the distribution of review authors' judgements across studies for each risk of bias item. (b) A summary table of review authors' judgements for each risk of bias item for each study.

Among the included studies, 2 articles [2, 7] mentioned allocation concealment and described the concealment method in detail, while the remaining studies were only mentioned “randomized.“ One study [2] was double-blind, while the remaining studies were open-label trials or with informed consent forms signed by patients. There were four [10–13] studies with no withdrawals or loss to follow-up. According to the Cochrane Handbook for assessing risk of bias, 6 included studies showed low risk of bias, while 1 at high risk of bias. The specific evaluation items and results are shown in Figures 2(a) and 2(b).

### 3.4. Meta-Analysis Results

This meta-analysis finally included 7 studies involving 624 patients, including 314 patients in the experimental group and 310 patients in the control group. The outcome measures included VAS pain score, incidence rate of adverse reactions, response rate, sleep interference score, and clinical global impression of change. The final results showed that duloxetine was slightly more effective than that of gabapentin in relieving DPNP and in safety, with a statistically significant difference.

#### 3.4.1. VAS Pain Score

Seven studies (314 cases in the experimental group and 310 cases in the control group) [2, 7, 9–13] compared the VAS pain score between the groups. The analysis results showed (SMD = −0.26, 95% CI: −0.53–0.002), *P* = 0.07) a slightly better outcome in the experimental group compared with the control group.

**Figure 3:**
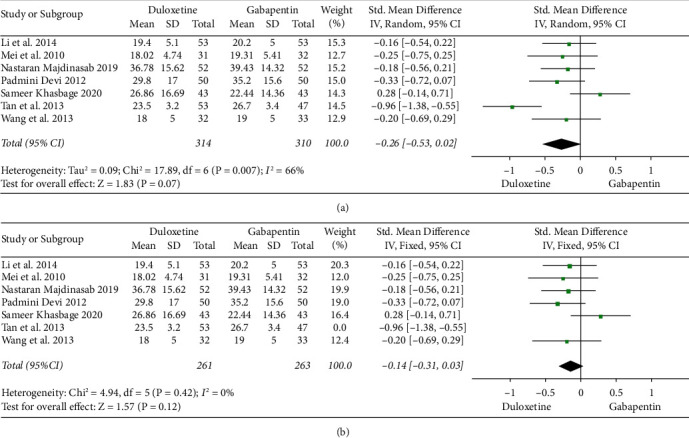
Forest plots of VAS pain scores of patients with diabetic peripheral neuropathic pain treated with duloxetine. (a) Forest plot containing all studies. (b) Forest plot with removal of Tan et al. [12].

In case of high heterogeneity (*P* = 0.007 and I^2^ = 66%), the random effects model was used to pool the effect sizes (Figure 3(a)), and sensitivity analysis was then performed to check whether the results were robust. By using one-by one elimination and after removal of the study by Tan et al. [12], the experimental group showed a slightly better VAS score than the control group (SMD = −0.14, 95% CI: −0.31–0.03, *P* = 0.12), and heterogeneity was significantly reduced (*P* = 0.42 and I^2^ = 0%) (Figure 3(b)).

#### 3.4.2. Incidence of Adverse Reactions

**Figure 4:**
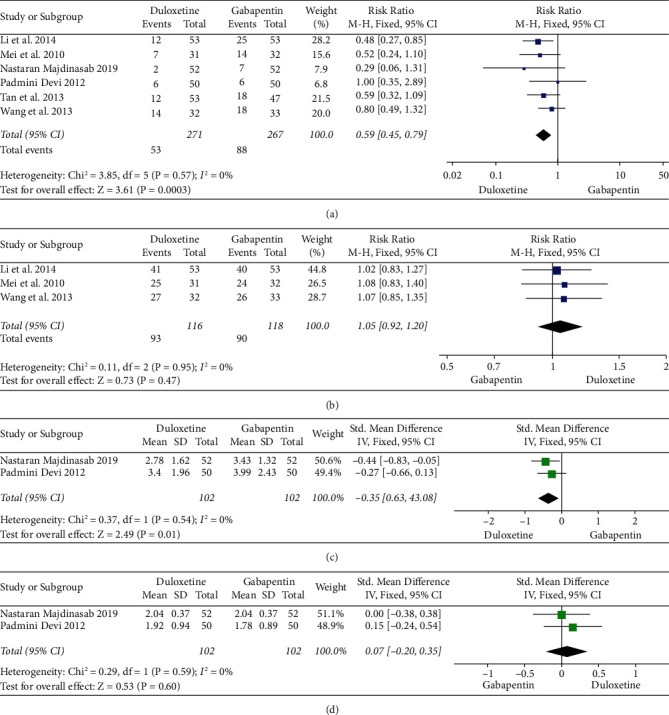
Forest plots of effect of duloxetine treating patients with diabetic peripheral neuropathic pain. (a) The incidence of adverse effects. (b) The response rate. (c) The sleep interference scores. (d) Clinical overall impression changes.

Six studies (271 cases in the experimental group and 267 cases in the control group) [2, 9–13] compared the incidence of adverse reactions between the groups, while one article [7] only reported the number of patients with each adverse reaction but no overall incidence of adverse reactions and therefore was excluded. The meta-analysis result of the six studies showed a lower incidence of adverse reactions in the experimental group compared with the control group (RR = 0.59, 95% CI: 0.45–0.79, *P* = 0.0003) (Figure 4(a)). No significant heterogeneity among studies was identified (*P* = 0.57, I^2^ = 0%), so the fixed effects model was used to pool the effect size.

#### 3.4.3. Response Rate

Three studies [10, 11, 13] compared the response rates between groups (116 cases in experimental group and 118 cases in control group). The studies by Wang et al. [13] and Mei et al. [11] only recorded the response rate in the control group until the second week, so all three studies only compared the response rate in the second week. The analysis results showed that the response rate in the control group was slightly higher than that in the control group (RR = 1.05, 95% CI: 0.92–1.20, *P* = 0.47) (Figure 4(b)). No marked heterogeneity was found (*P* = 0.95, I^2^ = 0%), so the fixed effects model was applied for combined analysis.

#### 3.4.4. Sleep Interference Score

Two studies [2, 9] reported the sleep interference score in the two groups. Since the course of treatment was 8 weeks in the study by Majdinasab et al. [2] and 12 weeks in that by Devi et al. [9], the sleep interference score at 8 weeks was uniformly calculated. The results showed that the sleep interference score at 8 weeks in the experimental group was lower than that of the control group (SMD = -0.35, 95% CI: −0.63 to −0.08, *P* = 0.01) (Figure 4(c)). The fixed-effects model was utilized to pool the effect size (*P* = 0.54 and I^2^ = 0%).

#### 3.4.5. Clinical Global Impression of Change

Two studies [2, 9] recorded the clinical global impression of change at 8 weeks after treatment in the two groups. According to the result, the experimental group showed a slightly better clinical global impression of change compared with the control group (SMD = 0.07, 95% CI: −0.20–0.35, *P* = 0.6) (Figure 4(d)). Low heterogeneity between studies was identified (*P* = 0.59 and I^2^ = 0%), so the fixed effects model was used for analysis.

### 3.5. Publication Bias

**Figure 5:**
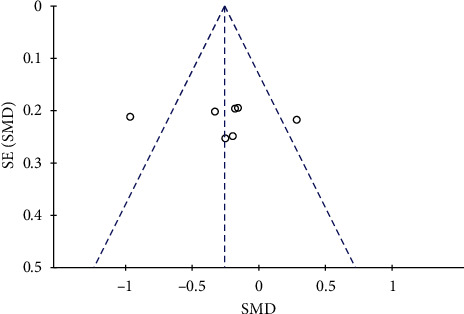
Funnel plot of VAS pain scores in patients with diabetic peripheral neuropathic pain treated with duloxetine.

The publication bias of this meta-analysis was examined by using funnel plots. Except for the VAS pain score, there were a small number of studies regarding the other outcome measures. Therefore, only the funnel plot of VAS score was analyzed. The results showed (Figure 5) that all the 7 studies [2, 7, 9–13] were in the funnel plot. The funnel plot was slightly skewed to the right, indicating a certain publication bias.

## 4. Discussion

DPNP is a common diabetic peripheral neuropathy, and its pathogenesis is related to the changes of ion channels on nerve cells, the role of glial cells in transmission of pain signals, and the central nervous system. DPNP not only cause pain and reduction of quality of life but also easily leads to problems such as diabetic foot ulcers, deformed foot, and consequently, increase of injury. Treatment for DPNP is usually based on control of basal blood glucose combined with SIRI drugs [14–17]. 5-HT and NE are descending inhibitory pathways of pain signal transmission present in the brainstem. 5-HT has the effects of ascending inhibition of pain perception and descending facilitation of pain perception; NE can only inhibit pain perception through alpha-2 adrenoceptors. SIRI drugs can enhance descending inhibitory pathway and reduce ascending of nociceptive signals and consequently achieve analgesia [18–20]. In addition, there are many overlaps in the pathogenesis of chronic pain and depression, so many anxiolytic and antidepressant drugs can also treat pain. The antidepressant duloxetine and the anticonvulsant gabapentin have been recommended for DPNP in the Evidence-Based Guideline: Treatment of Painful Diabetic Neuropathy which published on April 11, 2011, by the American Academy of Neurology (AAN), the American Association of Neuromuscular and Electrodiagnostics, and the American Academy of Physical Medicine and Rehabilitation.

Duloxetine, a SIRI drug, can increase 5-HT and NE concentrations in the brain and spinal cord and enhance the role of these two neurotransmitters in emotional regulation and sensitivity to pain, thus improving the body's tolerance to pain and consequently reducing and relieving pain. Gabapentin is a novel antiepileptic that inhibits formaldehyde and carrageenan-induced nociceptive processes and inhibits mechanical hyperalgesia and mechanical/temperature-related allodynia; it blocks neuropathic pain by acting on postsynaptic calcium channels in the spinal dorsal horn neurons.

This meta-analysis comprehensively compared the efficacy and safety of duloxetine and gabapentin in the treatment of DPNP using the following outcome measures: VAS score, response rate, incidence of adverse reactions, sleep interference score, and clinical global impression of change impression. After analysis, it was found that duloxetine was significantly superior to gabapentin in terms of the incidence of adverse reactions and sleep interference score, and there was no significant difference in terms of VAS score, response rate, and clinical global impression of change. Collectively, both duloxetine and gabapentin were effective for DPNP, but duloxetine was significantly superior to gabapentin in terms of safety. In in vitro experiments, duloxetine has shown a highly selectivity and has no significant affinity for about 60 neurotransmitter receptors (including dopamine receptors, adrenoceptors, cholinergic receptors, opioid receptors, glutamate receptors, and GABA receptors) nor for sodium channels, potassium channels, and calcium channels. Unlike other SIRI drugs, duloxetine does not inhibit monoamine oxidase [21], thereby reducing the incidence of adverse reactions and showing higher safety than gabapentin. Additionally, compared with gabapentin, it is more convenient for patients to take duloxetine (one capsule (60 mg)/day, oral medicine) [2, 7, 10–13], which further promotes the safety of duloxetine.

Notably, the results of our meta-analysis are in agreement with the previous systematic review published by Ko et al. [22] in terms of VAS score, suggesting that duloxetine compared to gabapentin had the similar efficacy in alleviating diabetic peripheral neuralgia. However, our meta-analysis incorporated evidence from additional studies, finding that duloxetine was superior to gabapentin in terms of safety. The meta-analysis of Ko et al. including three studies (*n* = 290) assessed the safety of both drugs by sleep disturbance scores and incidence of adverse reactions, concluding that there was no significant difference in safety. However, our study included seven studies (*n* = 624) that included evidence from China, which currently has the largest number of individuals of diabetes mellitus in the world [23] and where diabetic peripheral neuralgia is a common complication in patients with diabetes mellitus. Meanwhile, our study contained a larger sample size, assessing the safety of the two drugs by sleep disturbance scores and incidence of adverse reactions, yielding the final conclusions that duloxetine was better than gabapentin in sleep disorder score and incidence of adverse reactions.

This meta-analysis strives to be comprehensive and accurate, but there are still some limitations. First, the number of included studies and sample sized are small, which affects the robustness of the conclusion. Second, the study subjects are all from Asian countries but no populations from European, American and African countries, which limiting the application scope of the conclusion. Third, the maximum course of treatment in the trials is only three months but no trials with long treatment cycle. Fourth, there are few studies mentions some outcome measures (such as response rate, sleep interference score, and clinical global impression of change) in this analysis, so these outcome measures can only be used as secondary ones to supplement and explain the results. Fifth, funnel plot shows that there is a certain publication bias in this meta-analysis, which may be due to the insufficient number of included studies, hence the need for large-sample, multicenter studies to further improve the analysis results.

In summary, although this meta-analysis has limitations, it preliminarily confirmed that duloxetine has advantages in the treatment of DPNP, providing a reference value for the selection of medication. Duloxetine is worthy of clinical promotion and may have a promising application prospect due to its convenient medication, less side effects, and good safety.

## Figures and Tables

**Table 1:** The basic characteristics of the included studies.

Study	Year	Country	Study design	Medication methods/duration		Patients	Age	Baseline VAS	Outcomes
				Dul	Gab	Dul/gab	Dul/gab	Dul/gab	
Majdinasab et al. [2]	2019	Iran	Double-blinded, RCT	Duloxetine, 60 mg, QD/8 weeks.	Gabapentin, 300 mg, TID/8 weeks.	52/52	59.7 ± 5.6/60.7 ± 5.7	62 ± 21.2/64 ± 20	①②④⑤
Khasbage et al. [7]	2020	India	Open-label, RCT	Duloxetine, 60 mg, QD/12 weeks.	Gabapentin, 300 mg, QD/12 weeks.	43/43	53 ± 8.4/55.9 ± 10.8	72.44 ± 8.5/73.37 ± 10.56	①
Devi et al. [9]	2012	India	Open-label, RCT	Duloxetine, 120 mg, QD/12 weeks.	Gabapentin, 1800 mg, QD/12 weeks	50/50	58.48 ± 8.8/57.22 ± 10.5	57.1 ± 16.1/60.1 ± 17.6	①②④⑤
Wang et al. [13]	2013	China	RCT	Duloxetine, 60 mg, QD/4 weeks.	Gabapentin, 3600 mg, QD/4 weeks.	32/33	56 ± 5/55 ± 5	71 ± 12/71 ± 10	①②③
Tan et al. [12]	2013	China	RCT	Duloxetine, 60 mg, QD/8 weeks.	Gabapentin, 3600 mg, QD/8 weeks.	53/47	64.2 ± 7.9/64.7 ± 10.7	70.3 ± 7.8/72.4 ± 7.5	①②
Li et al. [10]	2014	China	RCT	Duloxetine, 60 mg, QD/4 weeks.	Gabapentin, 1200 mg, TID/4 weeks.	53/53	58.4 ± 12.5/57.6 ± 12.9	71.3 ± 10.9/71.7 ± 11.3	①②③
Mei et al. [11]	2010	China	RCT	Duloxetine, 60 mg, QD/4 weeks.	Gabapentin, 3600 mg, QD/4 weeks.	31/32	65.4 ± 5.2/55.2 ± 5.4	71.2 ± 11.2/70.4 ± 9.5	①②③

Dul : duloxetine group; Gab : gabapentin group; ①: VAS pain score; ②: incidence of adverse reactions; ③: response rate; ④: sleep disturbance score; ⑤: clinical overall change impression; RCT : randomized controlled trial.

## Data Availability

The data used to support the findings of this study are available from the corresponding author upon request.

## Abbreviations:

DPNP: Diabetic peripheral neuropathic pain
5-HT: Serotonin
NE: Norepinephrine
FDA: Food and Drug Administration
EFNS: European Federation of Neurological Societies
SNRI: Serotonin and norepinephrine reuptake inhibitor
GABA: Gamma-aminobutyric acid
NMDM: N-methyl-D-aspartate
RCTs: Randomized controlled trials
RR: Relative risk
CI: Confidence interval
SMD: Standardized mean difference
VAS: Visual Analogue Scale.
